# The Emerging Role of Mechanosensitive Piezo Channels in Migraine Pain

**DOI:** 10.3390/ijms21030696

**Published:** 2020-01-21

**Authors:** Adriana Della Pietra, Nikita Mikhailov, Rashid Giniatullin

**Affiliations:** 1 A. I. Virtanen Institute for Molecular Sciences, University of Eastern Finland, 70211 Kuopio, Finland; adriande@student.uef.fi (A.D.P.); nikita.mikhailov@uef.fi (N.M.); 2 Laboratory of Neurobiology, Kazan Federal University, 420008 Kazan, Russia

**Keywords:** Piezo channels, mechanotransduction, pain, migraine, CGRP

## Abstract

Recently discovered mechanosensitive Piezo channels emerged as the main molecular detectors of mechanical forces. The functions of Piezo channels range from detection of touch and pain, to control of the plastic changes in different organs. Recent studies suggested the role of Piezo channels in migraine pain, which is supposed to originate from the trigeminovascular nociceptive system in meninges. Interestingly, migraine pain is associated with such phenomenon as mechanical hypersensitivity, suggesting enhanced mechanotransduction. In the current review, we present the data that propose the implication of Piezo channels in migraine pain, which has a distinctive pulsatile character. These data include: (i) distribution of Piezo channels in the key elements of the trigeminovascular nociceptive system; (ii) the prolonged functional activity of Piezo channels in meningeal afferents providing a mechanistical basis for mechanotransduction in nociceptive nerve terminals; (iii) potential activation of Piezo channels by shear stress and pulsating blood flow; and (iv) modulation of these channels by emerging chemical agonists and modulators, including pro-nociceptive compounds. Achievements in this quickly expanding field should open a new road for efficient control of Piezo-related diseases including migraine and chronic pain.

## 1. Introduction

Touch, proprioception, and nociception are the fundamental senses mediated by activation of mechanosensitive ion channels, which are expressed in various types of sensory neurons. The detection of mechanical forces is mediated by different ion channels such as mechanosensitive ion channels (MSCs), potassium K2P channels, TMEM63/OSCA, and TMC1/2 [1]. In the current review, we will focus on the potential role of the recently discovered mechanosensitive Piezo1/2 channels in the nociceptive signaling in migraine. Migraine pain remains poorly understood mainly because of lack of mechanistic explanations for the initial steps in generation of pain signals in the nociceptive system. 

Migraine, a very common neurological disorder, is characterized by severe and long-lasting headache associated also with mechanical hypersensitivity and allodynia (pain induced by normally non-painful touch, which, during long-lasting migraine attack, is not limited to the head) [2]. Mechanosensitive ion channels most likely mediate mechanical hypersensitivity in the peripheral or central parts of the nociceptive system. However, nature of these ion channels remains unknown. Therefore, mechanosensitive Piezo channels, recently detected in human trigeminal ganglia [3,4], are the most probable candidates to mediate typical symptoms of migraine, such as mechanical hypersensitivity and pulsating type of migraine pain [5]. 

## 2. Complex Structure of Gigantic Piezo Channels

**Figure 1:**
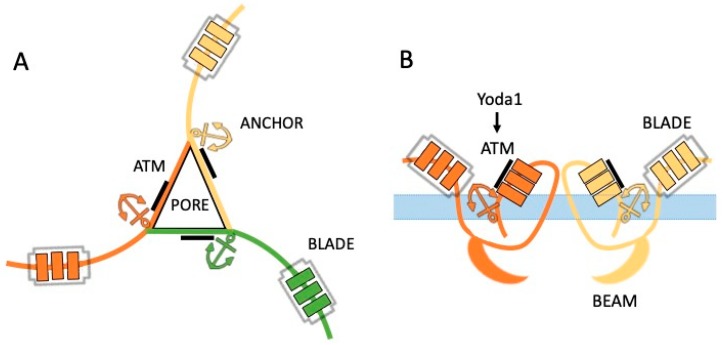
Schematic presentation of the Piezo channel. (**A**) Bird view of the Piezo channel with peripheral blade-like structures located in three subunits forming the trimeric functional unit with the central pore, blades and anchor regions. (**B**) Side view of the Piezo channel located in the lipid cell membrane. The following elements of the single subunit are presented: the intracellular beams, the C-terminals with the anchor regions and the ATM region containing the Yoda1 binding site and the extracellular blades.

Piezo channels are the family of mechanotransducers composed by two nonselective cationic channels known as Piezo1 and Piezo2 with a relatively homologous structure (Figure 1) [6,7,8,9]. Piezo channels are 2500 amino acids long proteins with up to 38 transmembrane segments per monomer. These monomers, combined together, build up the functional homo-trimers in the cell membrane [10]. Notably, these channels do not share sequence or structural homology with other above-mentioned mechanosensitive channels. This makes Piezo channels to be a completely new molecular target for therapeutic interventions, with their own profile of preferential physical triggers, chemical agonists, and specific modulators [11].

The molecule of Piezo1 channel is organized as a gigantic ‘three peripheral blade-like structure’ (Figure 1A), three 90-Å-long intracellular beam–resembling components bridging the blades together [10]. The central pore, formed by the C-terminals, has a main role in determining the channel conductance and ion selectivity [10]. The intracellular beam, part of the central cap, seems to be the perfect intermediate structure for mechanical transduction from the periphery to the central ion-conducting pore [7]. 

The uncommon structure of Piezo channels suggests their unique role in mechanical transduction [11], including various important functions in sensory neurons [12]. 

## 3. Functional Properties and the Role of Piezo1 vs. Piezo2 in Nociception

After discovery, isolation and cloning of Piezo1 and Piezo2 channels [8], the next challenging step would be to describe the functional properties of these channels in different cell types [9]. The natural activation of Piezo1 and Piezo2 channels can be achieved by different types of mechanical stimulation: stretching, pulling, pushing, exposure to hypo- or hyper-osmotic solutions, and flow-induced shear stress [8,13,14]. The kinetic properties of Piezo channels, studied by recording of the whole cell-inward currents in HEK cells, showed the fast activation and inactivation of these channels with recovery in the range of hundred milliseconds [6]. 

The comparison of two subtypes of Piezo channels showed that Piezo2 has a faster kinetics, and typically mediates a rapid membrane response, whereas Piezo1 channels are characterized by slower kinetics [8]. Thus, Piezo2 are more specified for detection of transient mechanical forces, whereas Piezo1 can react to more persistent activation [15]. Notably, these properties are sensitive to the channel’s environment in the membrane. Thus, the reduction of cholesterol in the cell membrane largely slowed down the inactivation kinetics of Piezo1 channels [16], which can support the persistence of signaling via this receptor type.

Consistent with fast kinetics, Piezo2 channels mediate the short-lasting mechanosensitive processes such as touch [6]. Piezo2 channels, implicated in this sensory modality, have been found in dorsal root ganglion (DRG) neurons [6]. Mechanical pain represents a different sensory modality, which is also expected to be mechanistically linked either to Piezo1 or Piezo2 channels if they are expressed in neurons mediating somatic and visceral pain. However, there are some controversies regarding expression of Piezo channel subtypes in nociceptive neurons. Indeed, one study showed the presence of Piezo2 but not Piezo1 channel in DRG neurons [17]. Another group [18] presented the evidence for the expression not only of Piezo2 but also of Piezo1 in DRG neurons. They found Piezo2 transcripts in sensory neurons of different sizes, including the largest diameter neurons mediating touch and proprioception. In contrast, Piezo1 was preferentially expressed in small size nociceptive neuron suggesting their role in pain [18]. Co-expression of Piezo1 and Piezo2 in the same neurons raises an interesting issue of functional interactions between these channels. The functional interplay between Piezo1 and Piezo2 channels was analyzed in the study where they found that the deletion of Piezo2 from the low-threshold mechanoreceptors in mice impaired touch but surprisingly sensitized mechanical pain, suggesting a negative interaction between Piezo1 and Piezo2 [19]. 

Our studies indicated that both Piezo1 and Piezo2 channels are expressed in trigeminal sensory neurons [5], which innervate head and face tissues and implicated in generation of migraine pain. Most of electrophysiological studies of Piezo channels in sensory neurons were performed by recording signaling from somas of these cells, representing a surrogate model of nerve terminals. However, more physiologically relevant approach to study the role of Piezo channels in migraine pain would be the recording of electrical spiking activity directly from the trigeminal nerve terminals in brain meninges [20]. Cranial meninges, comprising abundant blood vessels that are densely innervated by somatic and autonomous nerves, represent the so-called ‘trigeminovascular system’, which is considered as the origin site of primary headaches including migraine [21]. Application of the pro-inflammatory compounds to meninges induces mechanical sensitization of meningeal nociceptors [22], suggesting involvement of the professional mechanotransducers such as Piezo1 channels in this phenomenon. The repetitive nociceptive traffic in trigeminal neurons is a likely reason for mechanical hypersensitivity and allodynia, typical for migraine pathology [23]. Mechanical hypersensitivity can be directly linked to activation of Piezo channels in peripheral neurons whereas allodynia, mostly a central phenomenon, nevertheless, can also start from excessive and repetitive activation of peripheral Piezo channels in primary afferents. Taken together, these studies can serve as a background for the hypothesis on the role of Piezo channels in migraine. 

## 4. Unusual Chemical Activation of Piezo Channels

Since the discovery of Piezo mechanotransducers, the main tools to activate Piezo channels were the different types of mechanical stimulation. Unexpectedly, a very efficient alternative approach to activate Piezo channels has been recently found: that is the compound called Yoda1 [24]. The small lipid soluble molecule Yoda1 is able to activate specifically Piezo1 but not Piezo2 channels [24]. Yoda1 interacts with the C-terminal of the Piezo1 protein in the region of 1961–2063 amino acids, also known as the Agonist Transduction Motif (ATM) [25] (Figure 1B). The recent molecular dynamic simulations identified the Yoda1 binding pocket located in the domain approximately 40 Å away from the central pore [26]. Although the Piezo1 channel has three interacting monomers, interestingly, the binding of Yoda1 to only one subunit is already enough to open the ion channel [25], which provides a rationale for the high sensitivity of Piezo1 to this chemical agonist.

The other Piezo1 agonist, Jedi1/2, acts on the blade-beam structure inducing activation of the channel from the peripheral extracellular side [10]. However, it remains to be discovered if there are any endogenous molecules, which, similarly to the synthetic Yoda1 or Jedi1/2 compounds, can activate and/or sensitize Piezo1 channels in the healthy or disease states.

The discovery of chemical agonists of Piezo channels opened a new toolbox to investigate the function of mechanotransduction in different tissues, especially, when the traditional mechanical stimulation is not applicable or when the aim is to provide a widespread activation of Piezo channels in multiple targets. Thus, we found that the application of Yoda1 to the extended receptive field of meningeal afferents induced a massive and prolonged activation of trigeminal nerve fibers [5]. The nociceptive effect of Yoda1 in this study was reproduced by the similar prolonged activation of trigeminal mechanosensitive receptors by the hypo-osmotic solution [5]. Moreover, Yoda1 stimulation triggered the release of CGRP (calcitonin gene-related peptide) from these trigeminal nerve fibers. CGRP, the main migraine mediator, is known as a powerful promoter of meningeal inflammation and sensitization of trigeminal neurons [27,28,29]. 

These findings largely supported the proposed role of Piezo channels in peripheral mechanisms of migraine pain. Consistent with the pro-nociceptive role of Piezo channels activated by Yoda1 found in our study, Wang et al. [18] showed that Yoda1 induced a mechanical hyperalgesia with the prolonged time-course which is also typical for migraine pain. 

## 5. Puzzling Phenomenon of Pulsatile Pain: Role of Piezo?

The headache phase of a migraine attack is characterized by pulsating (throbbing) type of headache as a specific symptom of migraine pain [30]. This puzzling migraine phenomenon has been attracting attention of many migraine researchers and already served as a basis for the famous Wollf’s vascular theory of migraine headache [31]. This popular theory was supported by the clear correlation between the level of pulsations of the temporal artery and the intensity of headache during migraine attack in the patient treated with ergots [31]. Vascular mechanisms of migraine pain were also supported by the ability of the migraine mediator neuropeptide CGRP to produce the dilation of cranial vessels [32,33]. However, the role of vessels as the contributors to headache during migraine attack is still debated. As noted by Waeber and Moskowitz [34], the idea of abnormal dilatation of intracranial blood vessels leading to mechanical excitation of sensory fibers that innervate these vessels has never been validated. It is worth noting that there are also observations that the simple vasodilation of cranial vessels induced by the neuropeptide VIP is not enough to trigger migraine attack per se [35]. These findings added more intriguing aspects to the puzzling mechanisms of pulsating migraine pain. Notably, the ‘vascular theory’ suggested only the general explanation of pulsating migraine pain due to regular dilations of cranial vessels but it did not propose a molecular mechanism supporting this view. The discovery of Piezo mechanotransducers in trigeminal neurons suggested an attractive possibility to test these specific transducers as the sensors of the mechanical forces generated by pulsating vessels. Indeed, our recent investigation of Piezo channels in meningeal sensory nerve fibers allowed us to suggest a new model of mechanosensation in meninges during migraine attack [5]. 

## 6. New Model of Mechanosensation in Meninges during Migraine Attack

**Figure 2:**
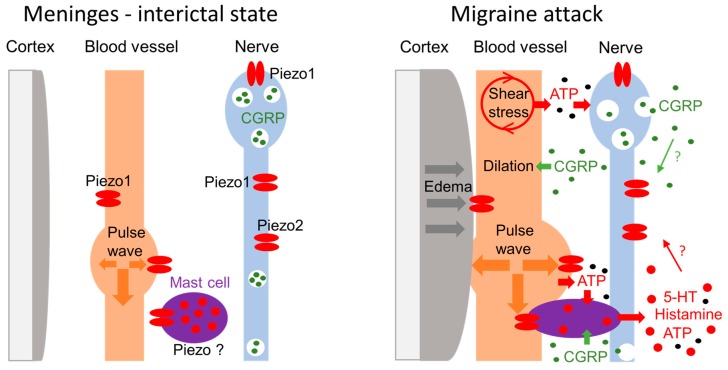
Schematic presentation of the key elements of the trigeminovascular system comprising meningeal blood vessels, local mast cells and trigeminal nerve fibers before and during migraine attack. **Left:** In the interictal state, before attack, there are only slight pulsations of meningeal vessels with the minimal activation of vascular Piezo1 channels or Piezo1 and Piezo2 channels in nerve fibers. **Right:** During migraine attack, which is often associated with brain oedema and CGRP-induced dilation of vessels, the extracellular space is reduced, promoting more close contact between pulsating vessels, nerves and nearby mast cells. The shear stress in dilated vessels and enhanced vascular pulsations promote mechanosensitive ATP release from the endothelial cells. Mechanical stimulation of calcium permeable Piezo channels in nerve fibers by pulsating vessels promotes neuronal CGRP release. CGRP and ATP can degranulate mast cells directly. In addition, the fraction of mast cells contacting vessels, is directly mechanically activated by blood pulsations. Activation of mast cells induces release of a plethora of pro-nociceptive compounds such as serotonin, histamine, leukotrienes, prostaglandins, ATP, and nitric oxide, further exciting the nociceptive fibers and promoting more CGRP release. All these pro-inflammatory compounds, in long run, together with CGRP, promote neuroinflammation, neuronal sensitization leading to long-lasting pulsating pain.

Based on the concept of the meningeal trigeminovascular system (TGVS) as the initial site for the generation of migraine headache [21], we propose the following model potentially explaining the mechanism of pulsating migraine pain. Figure 2 shows that in interictal state (or in healthy subjects), meningeal nerves express a plethora of pain transducing channels such as mechanosensitive Piezo1 and Piezo2 receptors [5], along with capsaicin-sensitive TRPV1 [36] and ATP-activated P2X3 receptors [37], which all are in low-active non-sensitized state. The main key components of the TGVS, such as meningeal vessels and neighboring nerves, in this state, have a low chance to interact to each other either physically or chemically and a low probability to generate pain signals.

The key event in migraine attack, is the release of the main migraine mediator CGRP [33], which has multiple actions in the TGVS (Figure 2, right). Thus, CGRP induces dilation of meningeal vessels, it promotes local neurogenic inflammation and degranulates dural mast cells, which, in turn, release several pro-inflammatory and pro-nociceptive compounds such as serotonin, histamine, ATP, prostaglandins, and nitric oxide [38,39,40]. Notably, most of these compounds are able to increase the sensitivity of meningeal afferents to mechanical stimuli [22,41]. Among other compounds released from mast cells, serotonin appeared to be the most strong and fast trigger of nociceptive spiking in nerve terminals [42,43]. Apart from the immediate firing of nociceptors, serotonin also promotes neuronal sensitization and local inflammation directly or through the additional release of CGRP [42]. The inflammation induced by CGRP, substance P and mediators of mast cells can sensitize not only peripheral but also central neurons, expanding the enhanced mechanical sensitivity to extracranial body regions, presented as the phenomenon of allodynia [22,44].

Notably, Piezo1 channels are expressed not only in neurons but also in vessels. Vasodilatation, shear stress and enhanced pulse waves in dilated vessels can activate mechanoreceptors in endothelial cells, triggering, via pannexins, ATP release (Figure 2) [45]. ATP is a strong promoter of nociceptive firing in meninges by itself [37,43] but it is also a trigger of mast cell degranulation promoting further release of the pro-inflammatory and pro-nociceptive compounds to meninges. 

Approximately one third of migraine cases is represented by migraine with aura. The pathophysiological mechanism underlying migraine aura is a phenomenon called ‘cortical spreading depression’ (CSD) [46,47]. CSD is a wave of strong depolarization of cortical neurons and glial cells, leading to meningeal neurogenic inflammation, involving the neuropeptide CGRP and substance P, ATP, and the mast cells activation [41,48]. Brain oedema, associated with CSD [46,49] compresses the extracellular space, which assists in the formation of more close contact between blood vessels and meningeal nerve fibers. Furthermore, CSD can increase the brain volume and raise the intracranial pressure [46]. Taken together, these factors, along with strongly pulsating vessels, should facilitate activation of mechanosensitive channels, such as Piezo ones, in nerve fibers [5]. 

Since meningeal tissues are protected by the skull from the external mechanical forces, the only source for activation of mechanosensitive Piezo channels in meningeal nerves are internal triggers, such as pulsating dural vessels. These pulsating dilated vessels can provide the regular Piezo-mediated excitation of nerve fibers processed towards the high-pain centers and perceived as a pulsating migraine pain. Notably, in migraine condition, there is an increased expansibility of arteries [50], which is also consistent with our hypothesis as the factor supporting the increased amplitude of pulsating waves. The exaggerated mechanical sensitivity of the trigeminal nerves during migraine attack can explain also the painful sensitivity to slight movements of the head or to cough [41].

## 7. How to Alleviate Migraine Pain Through Piezo Channels?

Accumulating evidence suggest that Piezo channels represent an attractive new molecular target to block pathological pain conditions via inhibition of these membrane’s mechanotransducers. Likewise, the idea of targeting Piezo channel for the novel type of analgesia might be extended to migraine pain. Given the pro-nociceptive role of Piezo channels in activation of meningeal afferents [5], the attractive approach would be to alleviate migraine pain through the direct or indirect inhibition of Piezo channels in trigeminal neurons. The antagonists of Piezo channels are of special interest as they potentially can block excessive activation of nociceptors in migraine conditions. However, currently available ligands of Piezo channels (Table 1) cannot directly serve as potential medicines for this neurological disorder. This is because the list of blockers of Piezo channels is limited and most of them are not specific (Table 1). Thus, there are Piezo channel inhibitors, such as the antagonist Dooku1, the blockers neurotoxin GsMTx4 and gadolinium [51]. Unfortunately, Dooku1 has a partial blocking activity in some cell types, whereas GsMTx4 or gadolinium are not specific for Piezo channels [51]. The main issue is that even if the specific blocker of Piezo1 is found, Piezo1 channels are not limited to trigeminal neurons but expressed in other cell types such as vessels (as mentioned above). Therefore, for the translational purposes, in migraine pain the alternative promising approach would be to find out the functional partners of these mechanosensitive channels or accessory proteins, which control the function of Piezo proteins. Although a purified Piezo1 protein retains the channel activity when reconstructed in artificial lipid bilayers [52]; in the natural environment, Piezo channels are likely under control of membrane lipids and certain intracellular messengers. The beforementioned functional interactions between Piezo1 and Piezo2, in trigeminal neurons, are of special interest but the underlying mechanism of such interactions requires further investigations. Likewise, little is known so far about the intracellular modulatory pathways for Piezo channels. However, it has been shown that, in response to phospholipase C (PLC) activation, both Piezo1 and Piezo2 channels could be inhibited by depletion of plasma membrane phosphoinositides [53]. The activation of PLC was supported by calcium influx via TRPV1 receptors, which are highly expressed in meningeal trigeminal afferents [36]. This finding suggests a negative functional crosstalk between Piezo and TRPV1 channels, which both are enriched in meningeal nociceptive fibers generating migraine pain [5,36]. Thus, our data showed that a fraction of trigeminal nerve fibers in meninges responded by nociceptive firing both to the TRPV1 agonist capsaicin and to the Piezo1 agonist Yoda1 [5]. The latter fact suggests that in trigeminal afferents in meninges there is a specific profile of nociceptive receptors, which can functionally interact. In contrast, in DRG sensory neurons, which mediate somatic pain, Piezo1 has a limited co-expression with TRPV1 channels [18].

An interesting family of potential Piezo modulators has been identified in the study showing that margaric acid, a saturated fatty acid that makes membrane stiffer, inhibits Piezo1 channels, whereas the poly-unsaturated fatty acid docosahexaenoic acid delays the inactivation of these channels [16] (Table 1). A dietary strategy to diminish the increased activity of Piezo channels in hemolytic anemia suggested by these authors could be extended to the excessive nociception via Piezo channels in migraine pain states associated with mechanical hyperalgesia. In particular, food enriched by margaric acid or other similarly actin fatty acids looks like a promising approach for these aims. 

Another open question, which has a translational perspective in migraine, is whether Piezo channels are modulated by the migraine mediators such as CGRP, serotonin, or NO. One study proposed the role of Epac1–Piezo2 axis in sensory neurons for the development of mechanical allodynia during neuropathic pain [54]. Epac, known as the sensor of cAMP, contains the conserved cAMP-binding domain and the level of cAMP can be raised in trigeminal neurons by the main migraine mediator CGRP [27,33]. However, all these pathways, activated by migraine mediators, which potentially can control Piezo channels, were not studied yet in migraine pathology.

Taken together, the pro-nociceptive Piezo channels in the trigeminovascular system represent a new promising target for the therapeutic interventions in migraine. Thus, the analgesic effect in migraine pain, can be approached either by the targeted delivery to the TGVS of the novel potent Piezo blockers, or by the inactivation of these mechanotransducers by modulators acting via changes in the lipid membrane environment. 

## 8. Summary and Outlook

Migraine is a common neurological disorder with many intractable cases. Despite the many decades of active investigations of this disorder, the molecular mechanisms implicated in the initial steps of migraine pain remain unclear. The recent studies suggesting Piezo channels as the vascular and neuronal sensors of intracranial mechanical forces present a new view on molecular processes implicated in meningeal nociception leading to migraine headache. These mechanosensitive mechanisms can underlie the worst migraine’s symptoms such as mechanical hyperalgesia and pulsating pain. Identification of novel molecular targets in meningeal trigeminovascular nociceptive system such as Piezo channels suggests the new approach to control this devastating neurological condition. 

## Figures and Tables

**Table 1:** Main agonists, antagonists and modulators of Piezo1 channels.

Agonists		Antagonists			Modulators	
Yoda1	Jedi1/2	Dooku1	Gadolinium	GsMTx4	Margaric acid (saturated)	Docosahexaenoic acid (unsaturated)
Selective		Selective	Nonselective		Nonselective	
Acting sites						
ATM region in C-terminus [24,25]	L15-16/L19-20 regions [10]	Yoda1 binding site [51]	Ion channel pore [51]		Changes in membrane lipid environment [16]	
					accelerated inactivation	reduced inactivation

## Abbreviations

MSCs	Mechanosensitive ion channels
K2P	Two-pore-domain potassium channels
TMEM63	Transmembrane protein 63
OSCA	Hyperosmolality-gated calcium-permeable channels
TMC1/2	Transmembrane channel-like protein 1/2
DRG	Dorsal Root Ganglion
CSD	Cortical spreading depression
CGRP	Calcitonin gene-related peptide
NO	Nitric Oxides
Epac	Exchange protein activated by cAMP
cAMP	Cyclic Adenosine Monophosphate
TGVS	Trigeminovascular system
